# Extensional Magnetorheology of Viscoelastic Human Blood Analogues Loaded with Magnetic Particles

**DOI:** 10.3390/ma14226930

**Published:** 2021-11-16

**Authors:** João M. Nunes, Francisco J. Galindo-Rosales, Laura Campo-Deaño

**Affiliations:** 1 CEFT, Departamento de Engenharia Química, Faculdade de Engenharia da Universidade do Porto, Rua Dr. Roberto Frias, 4200-465 Porto, Portugal; joaomiguelfn@sapo.pt (J.M.N.); galindo@fe.up.pt (F.J.G.-R.); 2 CEFT, Departamento de Engenharia Mecânica, Faculdade de Engenharia da Universidade do Porto, Rua Dr. Roberto Frias, 4200-465 Porto, Portugal

**Keywords:** magnetorheology, capillary thinning, extensional rheometer, blood analogues, magnetic particles

## Abstract

This study represents a pioneering work on the extensional magnetorheological properties of human blood analogue fluids loaded with magnetic microparticles. Dynabeads M-270 particles were dispersed in Newtonian and viscoelastic blood analogue fluids at 5% wt. Capillary breakup experiments were performed, with and without the influence of an external magnetic field aligned with the flow direction. The presence of the particles increased the viscosity of the fluid, and that increment was larger when embedded within a polymeric matrix. The application of an external magnetic field led to an even larger increment of the viscosity of the working fluids, as the formation of small aggregates induced an increment in the effective volume fraction of particles. Regarding the liquid bridge stability, the Newtonian blood analogue fluid remained as a Newtonian liquid exhibiting a pinch-off at the breakup time in any circumstance. However, in the case of the viscoelastic blood analogue fluid, the presence of the particles and the simultaneous application of the magnetic field enhanced the formation of the beads-on-a-string structure, as the Ohnesorge number remained basically unaltered, whereas the time of the experiment increased due to its larger viscosity, which resulted in a decrease in the Deborah Number. This result was confirmed with fluids containing larger concentrations of xanthan gum.

## 1. Introduction

Human blood is a complex fluid, composed of cellular elements like red blood cells (RBCs), white blood cells (WBCs) and platelets suspended in plasma, an aqueous solution (approximately 90–92 wt.% water) containing organic molecules, proteins and salts [1,2,3]. It is widely accepted by the scientific community that plasma exhibits a nearly Newtonian behavior [4,5]. In spite of the fact that WBCs and platelets could affect the rheology of whole blood, RBCs are responsible for the biggest influence, as they are present in a concentration of approximately 45% by volume in whole blood [1,3,4]. The presence of such a high volume of RBCs promotes the non-Newtonian character of blood, with varying shear-thinning viscosity, and thixotropic and viscoelastic properties [1,6].

A complete rheological characterization of the whole human blood is of great importance for the diagnosis and treatment of main cardiovascular diseases to understand and to plan drug delivery through the circulatory system, and for the design and development of medical equipment such as blood pumps, heart valves or stents, among others [7,8,9]. The use of micro- and nanoparticles to transport and separate materials, and to label and deliver therapeutic drugs to a target tissue [10] has caused a yearly increase in the studies in different fields of microbiology, biomedicine and biotechnology, leading to a growing need for an understanding of the rheological properties of human blood loaded with particles. The case of magnetic particles is of particular interest; they are very common in the field of biomedical applications, especially as carrier particles [11,12,13,14]. Due to their magnetic properties, they can be concentrated in the target zone by application of an external magnetic field, increasing the drug’s efficiency [14,15]. Blood loaded with magnetic particles is, by definition, a magnetorheological fluid, i.e. a magnetic field-responsive multi-phased system [16] consisting of magnetizable particles dispersed in a nonmagnetic liquid carrier.

Blood analogue solutions have been developed to replace real human blood for in vitro experiments. They possess characteristics such as nontoxicity, low cost and transparency, and avoid several complications regarding ethics, safety and costs [8,17,18]. These fluids are able to mimic the rheological behavior of human blood and are typically based on polymer solutions. Human blood and human blood analogues rheology were mostly assessed under shear flow [9]. While there are already some studies under extensional flow [8,19], Sousa et al. [9] suggest that whole blood rheology under extensional flow or in combined shear and extensional flows should be more deeply investigated to better understand the viscoelastic nature. There are already some studies about the capillary breakup of particulate suspensions, which verified that the presence of particles increases the bulk viscosity and, consequently, the whole thinning process of the liquid bridge is slowed down. However, close to breaking up, the filament thinning is accelerated due to the particle migration away from the thinnest part of the filament [20,21]. Extensional magnetorheology was also already applied in magnetorheological fluids and ferrofluids, concluding that the rheological properties of these fluids can be changed rapidly, reversibly and repeatedly when an external magnetic field is applied [22,23,24]. In the case of viscoelastic fluids, the rheological response under the application of a magnetic field is still unknown. To the best of the authors’ knowledge, no magnetorheological experiments under extensional flows with magnetic particles have been developed with viscoelastic fluids, where the elastic nature would play an important role. More specifically, there are no studies about the extensional magnetorheology properties of blood or blood viscoelastic analogues loaded with magnetic particles. Due to the current biomedical relevance of this system, this pioneering work may set the basis for a new research line in the field [25,26,27]. In this work, we focus on the assessment of the magnetorheological properties of blood analogue fluids loaded with magnetic microparticles under uniaxial extensional flow.

## 2. Materials and Methods

### 2.1. Blood Analogues

Newtonian and non-Newtonian (viscoelastic non-particulate) blood analogues were prepared and used for the extensional tests. An aqueous solution of 52 wt.% of dimethyl sulfoxide (DMSO) was prepared as a Newtonian blood analogue [17]. To address the viscoelasticity of blood a mixture of 100 ppm of xanthan gum (XG) in an aqueous solution of 52 wt.% of DMSO was formulated according to Campo-Deaño et al. (viscoelastic blood analogue) [18]. Another two extra solutions based on the same XG mixture were prepared with larger concentrations (250 and 500 ppm) in order to analyze the interaction between the particles and the polymeric matrix. 50 ppm of biocide sodium azide (SA) were added to all the solutions with XG to prevent bacterial growth, with no influence on their rheological properties [17]. The composition and properties of the working fluids are presented in Table 1 (XG from Sigma Aldrich, Algés, Portugal; SA from Riedel-de Haën, Seelze, Germany; and DMSO from CARLO ERBA Reagents, Sabadell, Spain). Densities were measured using a 5 mL pycnometer at ≈20 °C. Surface tension was also measured at ≈20 °C by means of a force tensiometer (Sigma 700 Biolin Scientific, Espoo, Finland) equipped with a Du Noüy ring of 0.185 mm in thickness and 9.58 mm in diameter.

### 2.2. Magnetic Particles

Magnetic particles, Dynabeads™ M-270 Carboxylic Acid (Thermo Fisher Scientific Inc., Vilnius, Lithuania) with a particle diameter of 2.75 µm and a density of 1.6 g/cm^3^ (Table 2), were considered for this study upon preliminary experiments (Tables S1 and S2, and Figures S1 and S2, Appendix A). They were dispersed at a fixed concentration of 5% wt. in all the solutions in Table 1.

For the discussion of the results, the magnetic characterization reported in Grob et al. [28] was assumed. The Dynabeads™ M-270 particles have a structure incorporating iron oxides (Fe_3_O_4_ or γ-Fe_2_O_3_) in a porous matrix, in a 14% wt. Regarding the magnetization curve, three regions may be distinguished: (a) A first region (H ≤ 5 kA/m), corresponding to weak external magnetic fields, where the microparticle magnetization is proportional to the applied magnetic flux density ($\vec{M}=\chi \vec{H}$); (b) a second region (5 kA/m ≤ H ≤ 40 kA/m), where the relation between the magnetization of the microparticle and the magnetic flux density is non-linear; (c) and, finally, a third region corresponding to the saturation region, where the microparticle magnetization converges towards the saturation magnetization ($M_{sat}$). In this case, a magnetic field of around 12 kA/m of intensity is applied, corresponding to the second region of the curve.

### 2.3. Extensional Rheometry and Extensional Magnetorheometry

The rheological characterization under extensional flow was performed in the Capillary Breakup Extensional Rheometer (HAAKE CaBER-1, Thermo Fisher Scientific, Waltham, MA, USA), equipped with 4 mm diameter plates (2R_0_). The initial aspect ratio is given by Λ0=h02R0; this is an important parameter to ensure reliable and successful results. It is required that h0lcap < 1, where lcap=Γρg=2.33 mm is the capillary length, to ensure that the interfacial force due to surface tension can keep the liquid bridge stable against the gravitational force. Previous numerical studies for filament stretching rheometry suggest that $\Lambda_{0}$ would be optimal in the range $0.5<\Lambda_{0}<1$ [29]. Considering all this, the initial height was set to 2 mm. The inertial effects during the filament thinning process have been minimized by means of the application of the Slow Retraction Method (SRM) [30].

**Figure 1:**
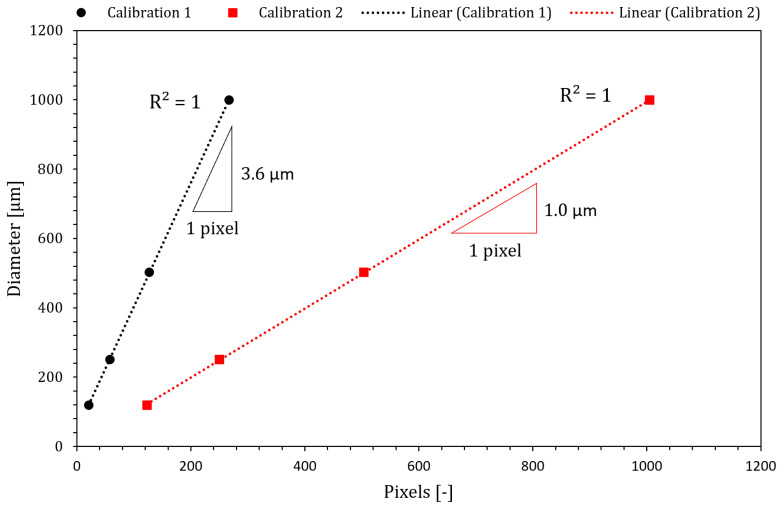
Calibration curves for the two optical configurations allowing recording at 20,000 fps with lower magnification (Calibration 1) and higher magnification (Calibration 2).

The silhouette of the filament thinning process was recorded by means of a high-speed camera (Photron FASTCAM Mini UX100, West Wycombe, Buckinghamshire, UK), equipped with a set of optical lenses (Optem Zoom 70 XL, Qioptiq, Fairport, NY, USA). A 52 mm Telecentric Backlight Illuminator provided a correct illumination and high contrast images of the liquid bridge profile, which was connected to a metal halide light source (LeicaEL6000, Leica Microsystems, Wetzlar, Germany) by means of an optical fiber cable, as detailed in recent works [24,31,32]. High-speed imaging in CaBER experiments induces less error in results than using the original laser micrometer [30,33]. The time evolution, t, of the filament thinning process was recorded using the camera in trigger-end mode at 20,000 fps from the moment of filament breakup (t_b_). In order to capture key information about the elastocapillary regime, the experiments were repeated, and the last part of the thinning process was recorded with higher magnification. Each set of images acquired during the course of each experiment was postprocessed in Matlab to detect the filament interface and determine the minimum diameter (2R_min_) along its axis of symmetry. Previously, the ratio micron/pixel was determined by means of recording a series of standard filament diameters (0.12, 0.25, 0.50 and 1 mm) with the optical setup at the same experimental conditions, providing resolutions of 3.6 µm/pix and 1.0 µm/pix (Figure 1). For each experiment here reported, more than three independent measurements were performed to ensure reproducibility.

The application of a constant magnetic field parallel to the extensional flow during the CaBER experiments was carried out by means of an apparatus developed by Galindo-Rosales et al. [23]. The fixture holds four rodlike permanent magnets centered at the corners of a square base with the fluid sample located at the center of symmetry of the prism. A homogenous magnetic field of 11.9 ± 0.1 kA/m is generated along the direction of the extensional flow (z-direction) [23]. This magnetic field would provoke a mild response in the magnetic particles, corresponding to the second region of the magnetization curve reported by Grob et al. [28]. In order to get a stronger response, the application of a magnetic field above 40 kA/m would be required (Figure S3, Appendix B).

To avoid particle sedimentation due to density mismatch between the particles ($\rho_{p}$ = 1.6 g/cm^3^) and the carrier liquid ($\rho_{l}$ ~1.07 g/cm^3^), once the sample was loaded in the CaBER the magnetic field was immediately activated, then the plates were separated and the filament thinning process recorded. All this procedure, including the duration of the filament thinning process, lasted less than 5 s, which is significantly smaller than the characteristic sedimentation time (~50 s).

## 3. Results and Discussion

### 3.1. Effect of Xanthan Gum Concentration

**Figure 2:**
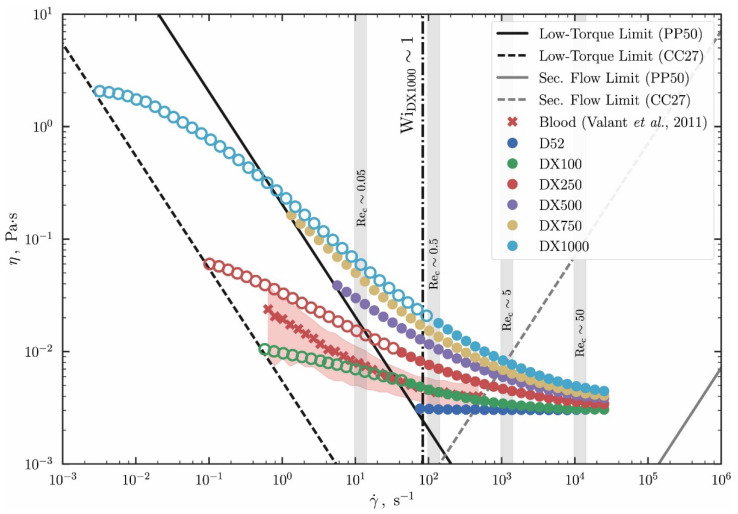
Viscosity curve of the XG solutions in water without the addition of magnetic particles. Reprinted from Journal of non-Newtonian Fluid Mechanics, 286, Rodrigues et al., Haemodynamics around confined microscopic cylinders, 104406, Copyright (2020), with permission from Elsevier. In this graph D52 is the Newtonian blood analogue (XG0 in the present manuscript). The addition of different concentrations of XG are referred to as DX100, DX250, DX500, DX750 and DX1000.

It has been already reported in previous work [17] that the steady-state shear viscosity curves of the working fluids without the magnetic particles deviates from the Newtonian behavior, exhibiting both an increase of the shear-thinning behavior and a slight increase in the infinite viscosity value ($\eta_{\infty }$), as the concentration of XG increases (Figure 2).

**Figure 3:**
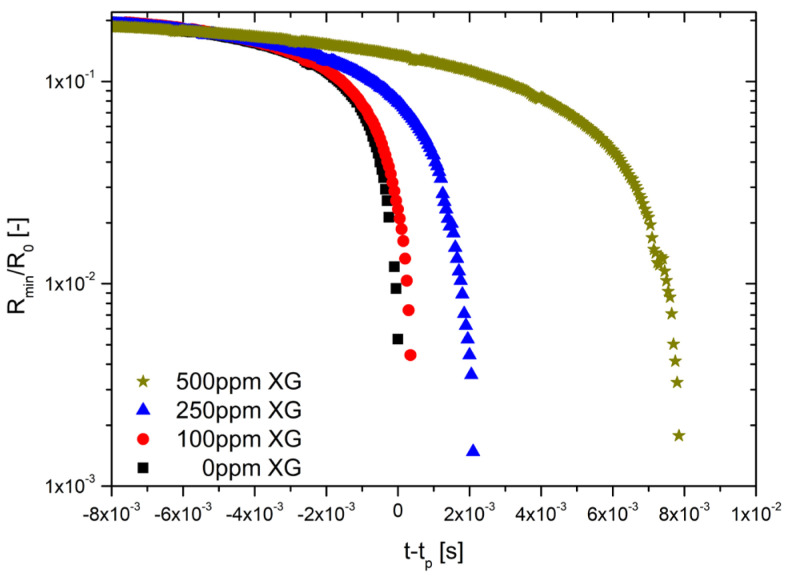
Effect of XG concentration on the breakup time of formulations XG0, XG100, XG250 and XG500.

The results from CaBER experiments for formulations not loaded with magnetic particles are shown in Figure 3, where the time evolution of the normalized filament radius R_min_/R_0_ is shown for the different XG concentrations. In this figure, the curves are shifted by the pinch-off time (t_p_) along the abscissa axis so that the initial potential flow regimes collapse onto the XG0 curve (which corresponds to the pure solvent). This behavior is normally noticed in low viscous fluids where the capillary pressure is only resisted by the inertia of the accelerating fluid molecules. On the contrary, for high enough extension rates ($\mathrm{Wi}=\dot{\varepsilon }\lambda$ > 0.5) the chains will eventually undergo a coil-stretch transition, start to unravel and begin to balance with their action of surface tension. At the point at which t−t_p_ = 0, there is a transition from the initial power flow thinning regime to an elasto-capillary balance regime and the necking fluid filament is formed into a long thin thread that thins exponentially with time. For the lowest concentrated sample (XG100), the minimum filament radius is initially moving towards the end-drops and the thin filament formed (stabilized by the unravelling polymer) is located on both sides between a large satellite drop in the middle and the two end-drops [30].

**Figure 4:**
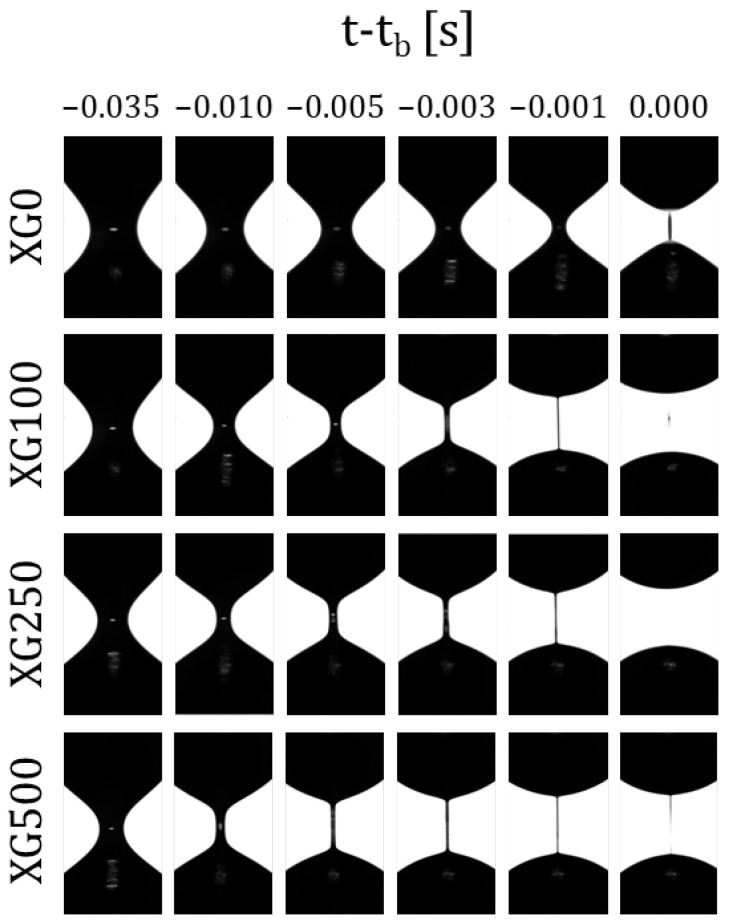
Last 35 ms of each filament thinning process for the different concentration of XG (0, 100, 250 and 500 ppm) at 22 °C.

Then, it was confirmed from Figure 2 and Figure 3 that the increase in the concentration of xanthan gum (XG) resulted not only in an increase in viscosity and a stronger shear-thinning behavior, but also in a significant increase in the elasticity and, therefore, in the relaxation time of the samples, as reported in Rodrigues et al. [17]. By analyzing the sequence of images shown in Figure 4, the increase in the elasticity of the samples with the increase in the XG concentration in the formulations is also noticeable, whereas XG0 showed a clear pinch-off at the moment of the breakup of the liquid bridge, characteristic of a Newtonian liquid; the samples containing XG exhibited a thread connecting the two end-drops, which is characteristic of viscoelastic fluids [34]. Within the elasto-capillary regime, the presence of the organic polymer results in normal stresses that counterbalance the capillary forces exerted by the surface tension; thus, the larger the concentration, the larger the normal stress; the filament lives longer and the relaxation and breakup times of the liquid bridge increase [34].

### 3.2. Extensional Magnetorheology of Human Blood Analogues Loaded with Magnetic Particles

**Figure 5:**
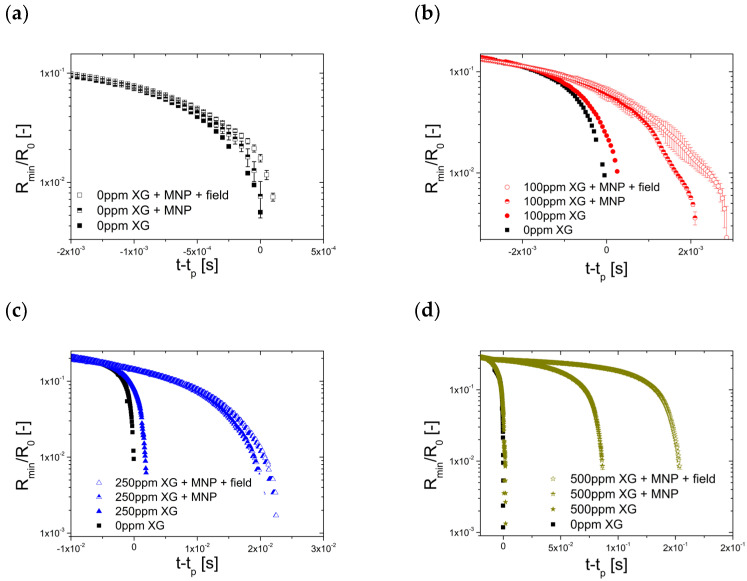
Effect of the addition of DB magnetic particles (MNP) and subsequent application of a magnetic field on the breakup time of formulations XG0 (**a**), XG100 (**b**), XG250 (**c**) and XG500 (**d**).

Figure 5 shows the time evolution of the normalized radius of the liquid filament for all the suspensions (with and without external magnetic field), and the time evolution of the minimum radius for the carrier fluid (XG0) for a better comparison of the effect produced by the magnetic particles.

In the case of the Newtonian analogue fluid (Figure 5a), the presence of the particles barely represented a delay in the breakup time, due to an increment in the viscosity of the fluids, as reported in [20,21] for other Newtonian fluids loaded with micron-sized particles. The presence of hard spherical particles in low concentration (diluted suspensions) represents an obstacle for the flow field and, therefore, local perturbations in the flow field are induced; this fact, together with the friction at the surface of the particle, results in an increase of the energy dissipation, and hence in viscosity, above that of the pure suspending medium. In other words, the suspension viscosity increases because the particles resist deformation and the internal stresses increase. Assuming the Dynabeads M-270 magnetic particles as rigid spheres and the concentration of 5% wt. as diluted (ϕ≅0.034), Einstein’s viscosity equation (Equation (1)) allows the estimation of the viscosity of the suspension (η) in the Newtonian analogue fluid [35]:

(1) $$\eta =\eta_{s}\left(1+2.5\phi \right),$$

where $\eta_{s}$ is the viscosity of the carrier fluid and ϕ is the volume fraction of particles. Thus, considering the particle concentration, an increase of 8.5% in the shear viscosity of the Newtonian analogue fluid can be estimated.

The application of the magnetic field (11.9 kA/m) slightly increases further the viscosity of the suspension in the Newtonian analogue fluid. While the magnetic field is not strong enough to induce a full magnetization of the particles and, consequently, a percolated network of particles cannot be generated, a mild magnetization is induced, resulting in magnetostatic forces that are strong enough to allow the formation of small clusters or aggregates of magnetic particles. These small clusters of particles are responsible for an effective volume fraction larger than the volume fraction calculated from the concentration ($\phi_{\mathrm{eff}}>\phi$) and, therefore, a larger distortion of the flow field is produced, resulting in an increase in the energy dissipation, and hence in the viscosity.

In the case of viscoelastic fluids, it has been experimentally [36,37] and numerically [38,39] demonstrated that the presence of particles at low volume fractions (ϕ<10%) induces a mild shear thickening behavior as a consequence of two mechanisms that change the suspension stress compared with the Newtonian counterpart: First, the stress contribution may change because the surface tractions change; second, there is an additional stress in the fluid phase due to the polymer stretching in the flow gradients induced by the particle. According to Einarsson et al., 2018 [40], when the viscoelastic suspension has undergone a simple shear flow, its shear viscosity ($\eta_{\mathrm{shear}}$) can be calculated by means of Equation (2):

(2) $$\eta_{\mathrm{shear}}=\eta_{s}\left(1+2.5\phi +\left(0.62-0.03\eta_{r}\right)\phi \eta_{r}{\mathrm{Wi}}^{2}\right),$$

where $\mathrm{Wi}=\underset{\gamma }{˙}\lambda$ is the Weissenberg number, $\underset{\gamma }{˙}$ is the applied shear rate, λ is the relaxation time of the polymeric solution and $\eta_{r}=\frac{\eta_{p}}{\eta_{s}+\eta_{p}}$ is the relative concentration of polymers given by the ratio between the solvent ($\eta_{s}$) and polymer ($\eta_{p}$) contributions to the shear viscosity $\eta_{0}$ = $\eta_{s}+\eta_{p}$ at Wi=0. Thus, for Wi=0 Equation (2) reduces to Equation (1). However, when the viscoelastic suspension is subjected to an extensional flow, the elongational viscosity ($\eta_{\mathrm{ext}.}$) is given by Equation (3):

(3) $$\eta_{\mathrm{ext}.}=3\eta_{0}\left(1+\eta_{r}\mathrm{Wi}+3\eta_{r}{\mathrm{Wi}}^{2}+2.5\phi +\phi \eta_{r}\left(2.68\mathrm{Wi}+9.36{\mathrm{Wi}}^{2}-0.1\eta_{r}{\mathrm{Wi}}^{2}\right)\right),$$

where $\mathrm{Wi}=\dot{\varepsilon }\lambda$; $\dot{\varepsilon }$ is the applied extension rate. It can be observed that in the limit of Wi=0 Equation (2) provides the Trouton Ratio ($\eta_{\mathrm{ext}.}=3\eta_{0}$). Moreover, Equations (2) and (3) provide an estimation of the shear and extensional viscosities as a function of the imposed shear and extensional rates, respectively, which has not been represented graphically here for the sake of clarity.

It can be observed in Figure 5b–d that the viscoelastic fluids loaded with Dynabeads M-270 particles without the application of an external magnetic field showed a marked influence on the time evolution of the minimum radius. The presence of the particles separates the curve from the curve corresponding to the unladen sample. According to Equation (3), the combined effect of shear-thinning behavior, elasticity and the volume fraction of the particles increases the resistance to the extensional flow, resulting in larger t-tp. Again, the application of a mild magnetic field aligned with the extensional flow favors the formation of aggregates that results in a larger effective volume fraction and, consequently, increases further the extensional viscosity and the curve separates a bit further from the case without the application of the magnetic field.

**Figure 6:**
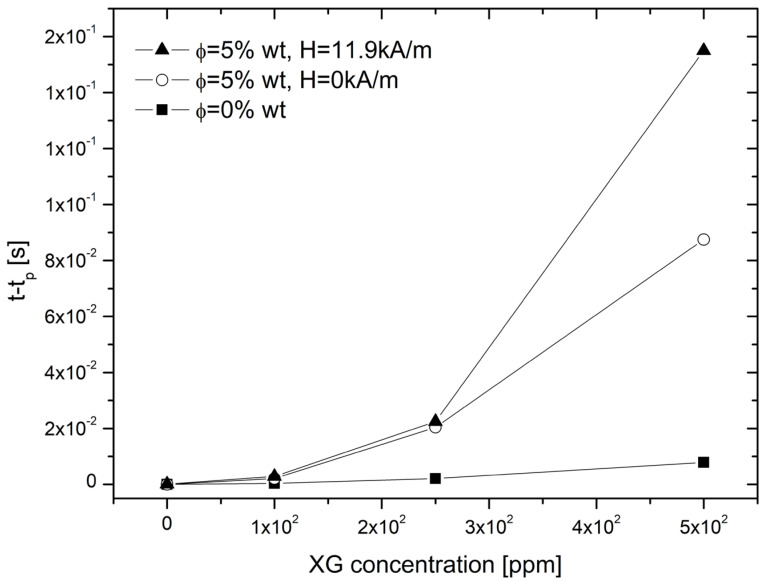
Dependence of the t-t_p_ at the breakup of the filament thinning process due to the presence of magnetic particles and magnetic field.

All the results discussed above can be summarized as in Figure 6. As $\eta_{\mathrm{ext}.}\propto t-t_{p}$, the interplay of the different parameters can be observed, i.e., the XG concentration, the concentration of magnetic particles and the application of an external magnetic field parallel to the extensional flow result in an increase of the extensional viscosity of the system. At a small XG concentration (<100 ppm), the lack of elasticity does not allow to increase the extensional viscosity by adding magnetic microparticles; however, for larger XG concentrations (>100 ppm), the extensional viscosity increases due to the presence of the particles, and even further when the magnetic field is applied, resulting from the formation of aggregates due to induced magnetostatic forces.

**Figure 7:**
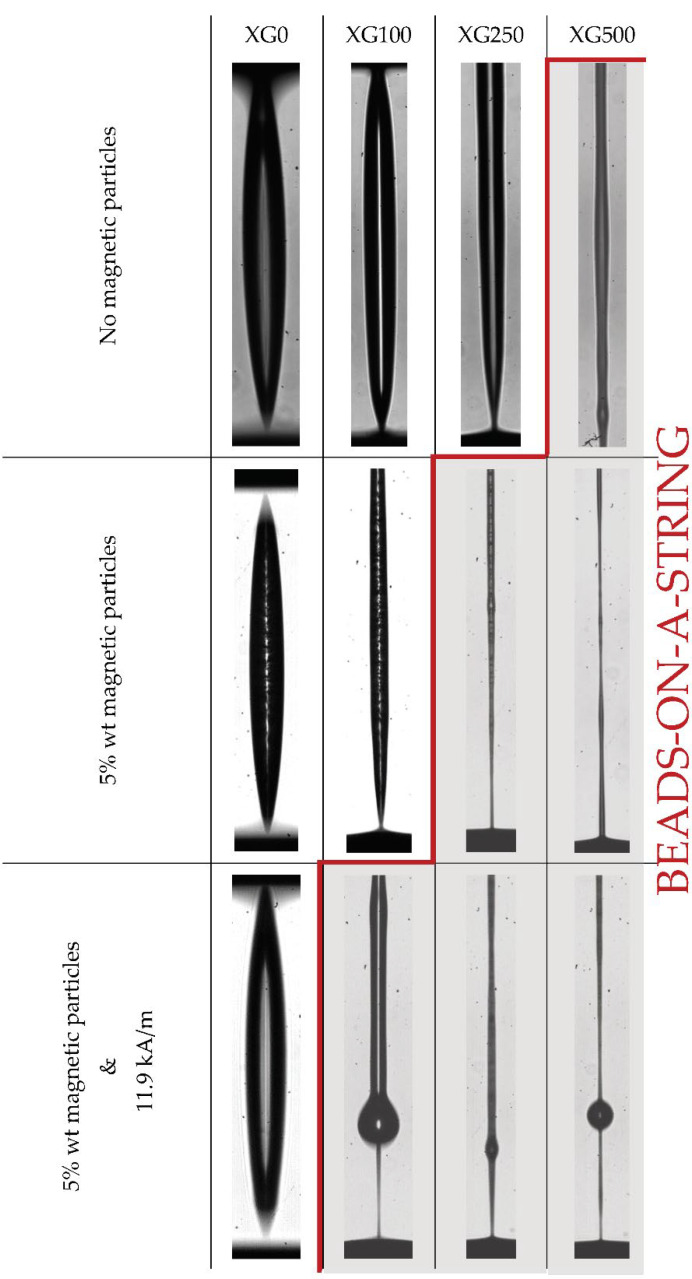
Filament silhouette of the different liquid filaments right before breakup. Beads-on-a-string structures are promoted by the presence of the particles under the external magnetic field.

Figure 7 shows a comparison between the shapes of the filament profiles right before breakup, depending on the XG concentration, the concentration of magnetic particles and the application of the external magnetic field aligned with the extensional flow. The axisymmetric nature of the filament provides an optical aberration due to the refractive index mismatch between the liquid sample and the surrounding air, which is responsible for the black lateral bands. At the maximum resolution (1 pix/μm) it is possible to infer the presence of the particles, but it was not possible to observe either the presence of aggregates or the microstructure formed when the magnetic field was applied. However, it was indeed possible to see that the particles did not migrate from the thinnest part of the filament, contrary to the observation reported for larger particles [20,21]. It can also be observed that in the Newtonian analogue fluid, the addition of magnetic particles and the application of the magnetic field may increase the viscosity of the samples, but they remain as Newtonian liquids exhibiting a pinch-off at the breakup time. The addition of XG adds a certain elastic nature to the sample and at the largest concentration the onset of beads-on-a-string (BOAS) formation was observed; the addition of particles and without the application of the magnetic field increases both the viscosity and the time of the experiment, and the onset of BOAS moves down towards 250 ppm of XG; finally, the application of the field increases further both the viscosity and the time of the experiment and promotes the formation of beads-on-a-string structures during the breakup process even at 100 ppm.

**Figure 8:**
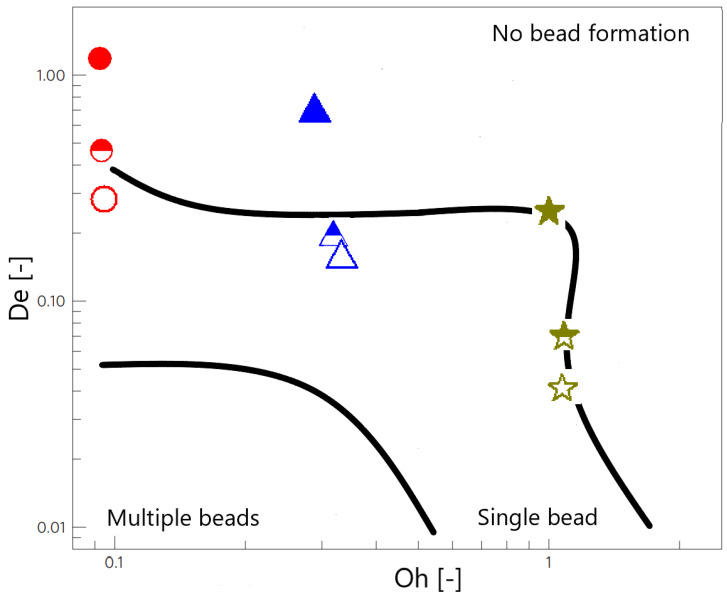
Phase diagram depicting the regions showing different BOAS morphologies in the De and Oh space adapted from [42]. Symbols represent the experimental data corresponding to this work, following the same color and symbol scheme as in Figure 5.

The beads-on-a-string structures consist of single or multiple spherical fluid drops (beads) interconnected by slender threads, and they are characteristic of viscoelastic liquids that have undergone a filament thinning process [41]. However, the formation of beads-on-a-string structures depends on a delicate balance between two dimensionless parameters: (1) The Ohnesorge number ($\mathrm{Oh}=\frac{\eta_{s}+\eta_{p}}{\sqrt{\rho \sigma R}}$), which relates the viscous force to inertial and surface tension forces; and (2) the Deborah number ($\mathrm{De}=\frac{\lambda }{\tilde{t}}$), which relates the relaxation time and the time scale of the experiment ($\tilde{t}$). Looking at the phase diagram (Figure 8) reported by Bhat et al. [42] it is possible to observe that the addition of the particles and the application of the magnetic field increased the lifetime of the experiment, resulting in a decrease in the De, whereas the Ohnesorge number remained sensibly unaltered for each polymer within the range in which inertial forces are still important, i.e., Oh < 1. As it can be observed, the prediction of the formation of BOAS is faithfully reproduced in the samples studied.

The breakup time is the time it takes for a liquid bridge to break under a step-strain extensional experiment in the CaBER-1 device and it is shown in Figure 6 that it increases non-linearly with the concentration. It has been reported in the literature [34] that the breakup time is the result of a force balance between the capillary forces that aim at breaking the liquid filament and the internal forces that aim at maintaining the bridge, which are a combination of viscous, inertial, elastic, gravitational and other forces. Thus, an increase in the polymer concentration would increase not only the viscosity but also the relaxation time, which is linked directly to the elastic force. Regarding the viscosity dependence with the concentration, a deviation from the linear dependence of the reduced viscosity has been reported with the concentration above a critical concentration; the behavior is then more complicated in the semi-dilute regime due to interpenetration of the macromolecular domains or entanglements [43]. It has also been reported in the literature that polymer solutions in dilute and semi-diluted regimes increase the relaxation time following a quadratic function of the concentration [44]. Considering this background information and the fact that the working liquids in this study are in the dilute and semi-diluted regimes [17], it is not surprising to see a non-linear evolution of the breakup time with the increasing concentration. As we report here, the presence of the particles induces the formation of beads-on-a-string structures (Figure 7), which are responsible for the remarkable enhancement of the lifetime of the filament observed in Figure 6, according to the work of Bhat et al. [41].

## 4. Conclusions and Remarks

This study represents a pioneering work within the frame of elucidating the effect of the dispersing magnetic microparticles when a simultaneous external magnetic field is applied on the rheological properties of blood. Rheological blood analogue fluids have been considered in this work.

The dispersion of Dynabeads M-270 particles in the working fluids resulted in an increment of the viscosity, which was larger when embedded within a polymeric matrix. That increment was even larger under the influence of the magnetic field, as the formation of small aggregates induced an increment of the effective volume fraction of particles.

In the case of the Newtonian blood analogue, it remained as a Newtonian liquid exhibiting a pinch-off at the breakup time in any circumstance. However, in the case of the viscoelastic blood analogue fluid (XG100), the presence of the particle and the application of an external magnetic field induced the formation of the beads-on-a-string structure. The same behavior was observed for larger concentrations of XG. These results are of paramount importance as they constitute clear evidence of the variation of the rheological properties of blood, especially in the cases of a high degree of elasticity that can be related to some blood diseases. This fact would help to find more precise diagnostic techniques. In future works, we will explore the influence of larger magnetic fields, different particle sizes and concentrations in the magnetorheological properties of blood.

## Figures and Tables

**Table 1:** Composition and properties of the working solutions.

Acronym	Formulation	Blood Analogue	Density [g/cm^3^]	Surface Tension [mN/m]
XG0	0 ppm XG + 52% wt. of DMSO in water	Newtonian [17]	1.070	56.95 ± 0.01
XG100	100 ppm XG + 52% wt. of DMSO in water	Viscoelastic [18]	1.071	56.63 ± 0.01
XG250	250 ppm XG + 52% wt. of DMSO in water	Viscoelastic [17]	1.071	57.30 ± 0.01
XG500	500 ppm XG + 52% wt. of DMSO in water	Viscoelastic [17]	1.072	57.01 ± 0.01

**Table 2:** Physical properties of Dynabeads™ M-270 (ThermoFisher) [28].

Magnetic Particle	Density [g/cm^3^]	Iron Content [% wt.]	Diameter [µm]
DB M-270	1.6	14	2.75

## Supplementary Materials

The following are available online at https://www.mdpi.com/article/10.3390/ma14226930/s1, Table S1: Comparison between the main properties of the magnetic particles used in this study. All data is provided by the manufacturers, except the diameter of carbonyl iron particles, which was taken from Sadek et al. [29]; Table S2: Composition and properties of the carbonyl iron suspensions; Figure S1: Effect of the addition of CIP (•-5%; ••-10% *w*/*w*) and subsequent application of a magnetic field (⊙) on the breakup time of formulations XG100 (A), XG250 (B) and XG500 (C); Figure S2: Temporal evolution of the filament profiles for different concentration of XG (100, 250 and 500 ppm), loaded with 5 % (•) and 10 % (••) *w*/*w* of CIP, at 22 °C. The formulations with suspended magnetic particles were also assessed under a magnetic field parallel to the flow (⊙); Figure S3: New add-on designed for the CaBER-1 device in order to perform extensional magnetorheometry under large magnetic fields: (a) sketch of the design and dimensions of the different parameters considered in the numerical analysis; (b–e) intensity of the magnetic field generated with permanent magnets of Neodymium N45 with a cylindrical shape having r = 2 mm and h = 3 mm; (f) intensity of the magnetic field in the axis of the magnets for different values of the distance between the magnets (H); (g) real picture of the add-on with the different components attached to the CaBER-1 device.

## Appendix A. Sedimentation Issues with Carbonyl Iron Particles

In the first batch of formulations loaded with magnetic particles, we used Carbonyl Iron Particles (CIP) in two different concentrations (Tables S1 and S2). These did not show significant rheological differences between them, for each XG concentration assessed (Figure S1). The extensional properties did not show either any significant differences when the magnetic field was applied. A slight increase in the breakup time of the filaments was verified in formulations loaded with magnetic particles, with and without magnetic field, compared to solutions without particles in suspension. The presence of particles increases the bulk viscosity and therefore delays the thinning process [20,21]. The magnetic field (11.9 kA/m) did not interfere with this effect. Figure S2 shows the temporal evolution of the filaments for different concentrations of CIP in the XG solutions (100, 250 and 500 ppm).

The weak rheological response after the addition of magnetic particles and the posterior application of a magnetic field can be justified by the rapid sedimentation velocity due to density mismatch between particles and suspending fluid. Since the sedimentation velocity is relatively high (≈3.0 mm/s) at the beginning of the experiment, the sedimentation time is of the order of 0.5 s; whereas the experiment time scale is of the order of 5 s; thus, it is highly possible that the iron particles did not hold enough time in suspension to produce noticeable rheological effects, even when magnetic field is applied.

## Appendix B. Increasing the Intensity of the Magnetic Field

In order to try magnetizing further the Dynabeads M-270 particles, a new setup was designed in order to apply a magnetic field with larger intensity values. Both endplates in the CaBER-1 rheometer were replaced by means of two permanent magnets with cylindrical shape, h = 3 mm and r = 2mm (Figure S3a). The distance between the magnets (H) controls the intensity and homogeneity of the magnetic field (Figure S3b–e). We have performed numerical simulations in COMSOL Multiphysics for H = {2, 3, 4, 5, 6} mm and results showed that the closer the distance between magnets, the more homogeneous was the magnetic field (Figure S3d). Figure S3f shows the physical aspect of the new add-on; each of 3D-printed plastic holders has two hollow parts, one for introducing the shaft of the CaBER-1 device and another one for fixing the cylindrical permanent magnet (3mm height and 2mm radius, Neodymium N45, nickel-plated, Supermagnete, Germany).

The initial gap for the experiments with this configuration was set again at 2 mm. According to the numerical results, it becomes obvious that as long as the top-plate separates from the bottom one, the intensity of the magnetic field decreases, and the field becomes inhomogeneous. In the worst-case scenario (6 mm separation and at the center of the fluid volume), the intensity of the magnetic field would be more than 10 times the intensity of the magnetic field provided with the apparatus used in Galindo-Rosales et al. [23]. This order of magnitude in the intensity of the magnetic field would allow reaching the saturation zone of the Dynabeads particles [28] and the magnetorheological response of the working formulations would have been more significant. However, the combined effect of having a strong magnetic field gradient along the z-direction and the high magnetophoretic mobility of the Dynabeads M-270 particles [28] does not allow magnetic particles to remain in suspension long enough to assess their effect on the extensional rheology of our formulations. Therefore, this setup turned out to be impractical, as the particles were glued immediately to one of the plates as soon as they were dispensed from the syringe. Further research on the development of a new magnetorheological cell for the CaBER is required.
