# Supplementary material for: Extensional Magnetorheology of Viscoelastic Human Blood Analogues Loaded with Magnetic Particles
Source: Materials (Basel). 2021 Nov 16;14(22):6930. doi: 10.3390/ma14226930 (PMC8621293; doi:10.3390/ma14226930)
Supplement: Supplementary file 1 [file materials-14-06930-s001.zip › materials-1438052-supplementary.pdf]

materials

Supplementary Materials

Extensional Magnetorheology of Viscoelastic Human Blood Analogues Loaded with Magnetic Particles

João M. Nunes [1], Francisco J. Galindo-Rosales [1] and Laura Campo-Deaño [2,*]

[1] CEFT, Departamento de Engenharia Química, Faculdade de Engenharia da Universidade do Porto, Rua Dr. Roberto Frias, 4200-465 Porto, Portugal; joaomiguelfn@sapo.pt (J.M.N.); galindo@fe.up.pt (F.J.G.-R.)
[2] CEFT, Departamento de Engenharia Mecânica, Faculdade de Engenharia da Universidade do Porto, Rua Dr. Roberto Frias, 4200-465 Porto, Portugal
* Correspondence: campo@fe.up.pt

Citation: Nunes, J.M.; Galindo-Rosales, F.J.; Campo-Deaño, L. Extensional Magnetorheology of Viscoelastic Human Blood Analogues Loaded with Magnetic Particles. *Materials* **2021**, *14*, 6930. https://doi.org/10.3390/ma14226930

Academic Editor: Yannis Dimakopoulos

Received: 12 October 2021
Accepted: 9 November 2021
Published: 16 November 2021

Publisher's Note: MDPI stays neutral with regard to jurisdictional claims in published maps and institutional affiliations.

Copyright: © 2021 by the authors. Licensee MDPI, Basel, Switzerland. This article is an open access article distributed under the terms and conditions of the Creative Commons Attribution (CC BY) license (http://creativecommons.org/licenses/by/4.0/).

Table S1. Comparison between the main properties of the magnetic particles used in this study. All data is provided by the manufacturers, except the diameter of carbonyl iron particles, which was taken from Sadek et al. [31].

Name.	Acronym	Diameter [μm]	Density [g/cm³]
Carbonyl iron particles	CIP	7.2 ± 4.6 [1]	7.86
Dynabeads™ M-270 Carboxylic Acid	DB	2.75	1.6

[1] Sadek et al. [31].

Table S2. Composition and properties of the carbonyl iron suspensions.

Acronym	Constitution	Density [g/cm³]	Surface Tension [mN/m]
XG100•	XG100 +5% wt. of carbonyl iron particles	1.112	57.293 ± 0.005
XG250•	XG250 +5% wt. of carbonyl iron particles	1.114	57.092 ± 0.003
XG500•	XG500 +5% wt. of carbonyl iron particles	1.122	57.360 ± 0.007
XG100••	XG100 +10% wt. of carbonyl iron particles	1.189	57.537 ± 0.005
XG250••	XG250 +10% wt. of carbonyl iron particles	1.195	57.642 ± 0.005
XG500••	XG500 +10% wt. of carbonyl iron particles	1.199	57.410 ± 0.007

•+5% wt; ••+10% wt.

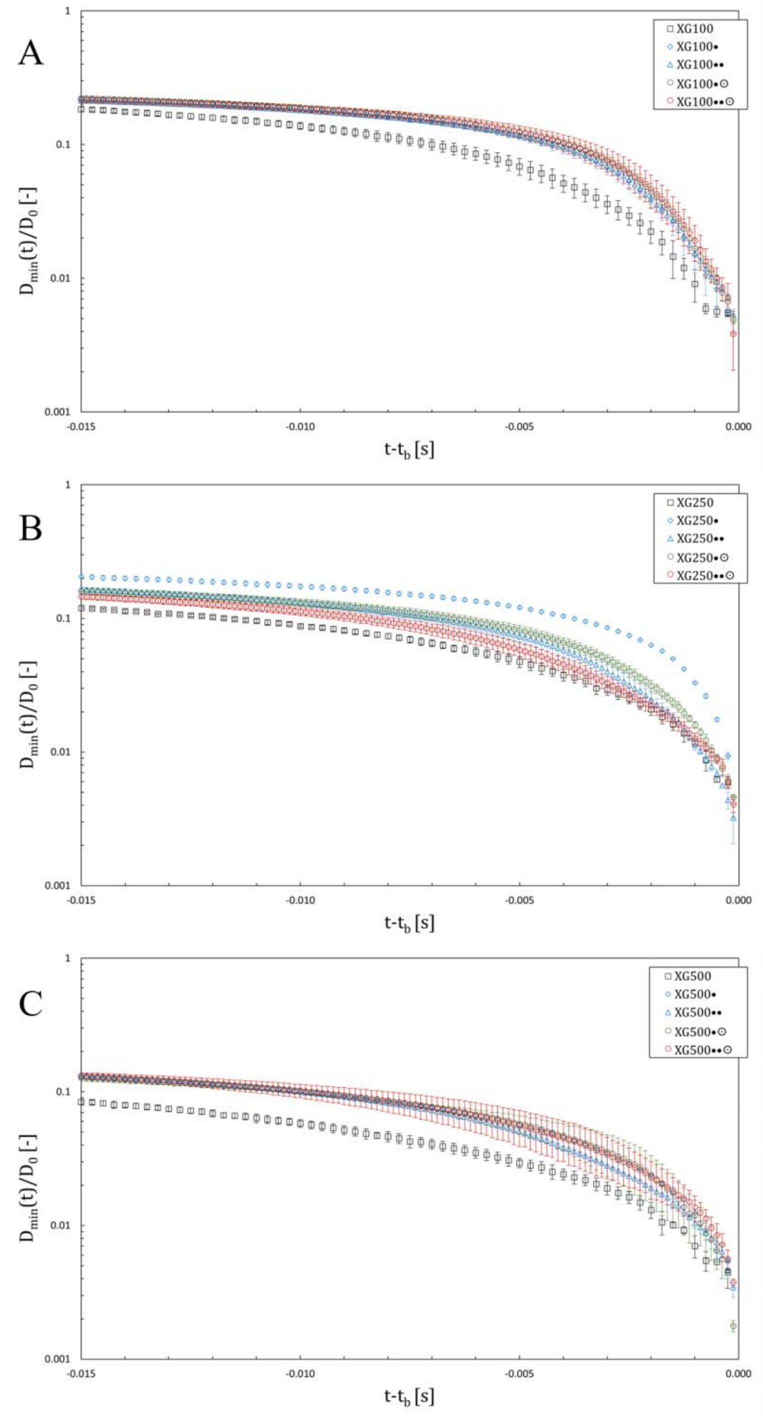

Figure S1. Effect of the addition of CIP (•—5%; ••—10% w/w) and subsequent application of a magnetic field (⊙) on the breakup time of formulations XG100 (**A**), XG250 (**B**) and XG500 (**C**). All points are presented with an error bar corresponding to the standard deviation of three assays.

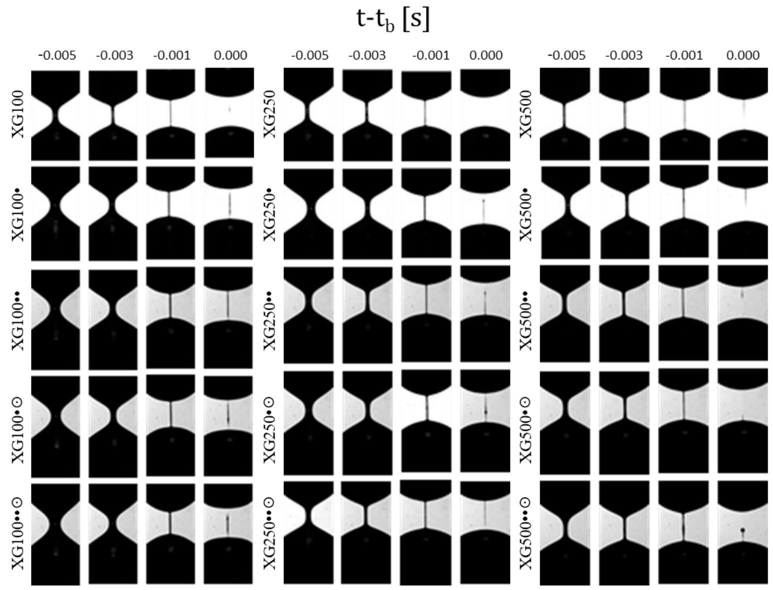

Figure S2. Temporal evolution of the filament profiles for different concentration of XG (100, 250 and 500 ppm), loaded with 5% (•) and 10% (••) w/w of CIP, at 22 °C. The formulations with suspended magnetic particles were also assessed under a magnetic field parallel to the flow (⊙).

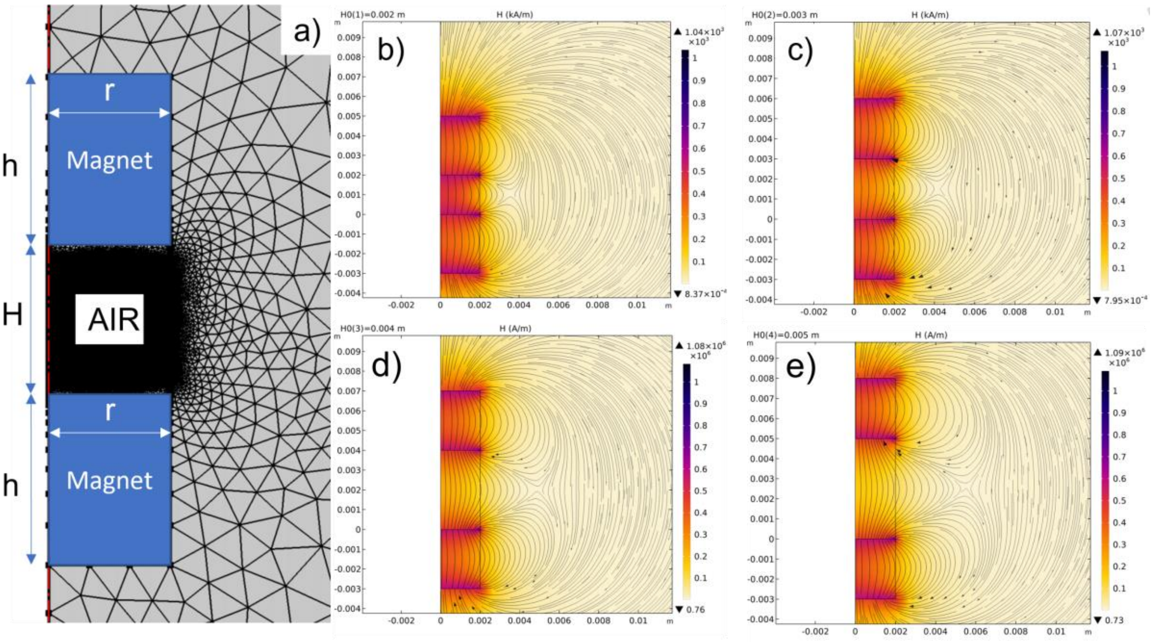

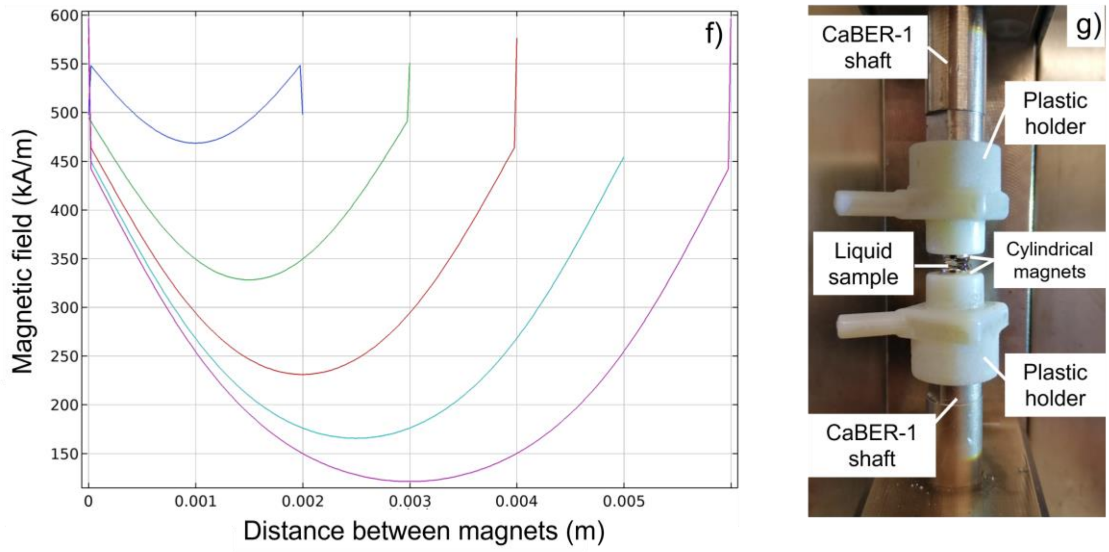

Figure S3. New add-on designed for the CaBER-1 device in order to perform extensional magnetorheometry under large magnetic fields: (**a**) sketch of the design and dimensions of the different parameters considered in the numerical analysis; (**b**–**e**) intensity of the magnetic field generated with permanent magnets of Neodymium N45 with a cylindrical shape having r = 2 mm and h = 3 mm; (**f**) intensity of the magnetic field in the axis of the magnets for different values of the distance between the magnets (H); (**g**) real picture of the add-on with the different components attached to the CaBER-1 device.
